# An alternative linear impactor for impact research

**DOI:** 10.1016/j.ohx.2026.e00742

**Published:** 2026-01-14

**Authors:** Poomkarn Taedullayasatit, Sitthichok Sitthiracha, Manus Dangchat, Nattawood Prasartthong

**Affiliations:** a Shrewsbury International School Bangkok Riverside, 1922 Charoen Krung Road, Wat Praya Krai, Bang Kholaem, Bangkok 10120, Thailand; b Rajamangala University of Technology Rattanakosin, 96, Moo 3, Phutthamonthon Sai 5 Road, Salaya, Phutthamonthon, Nakhon Pathom 73170, Thailand; c The Sirindhorn International Thai-German Graduate School of Engineering, King Mongkut’s University of Technology North Bangkok, 1518 Pracharat 1 Road, Wongsawang, Bangsue, Bangkok 10800, Thailand

**Keywords:** Linear impactor, Punch simulator, Test rig

## Abstract

This paper introduces an affordable and easily replicable linear impactor designed for impact research. Traditional commercial systems are often expensive and complex, limiting their accessibility to many research and development teams. Our hardware addresses this by providing a reliable platform for conducting controlled impact experiments. The system uses a spring-driven ram to strike an object equipped with instrumentation for measuring impact responses. The design allows for customization and integration with various test objects and setups, making it adaptable for evaluating different impact studies such as the effectiveness of protective devices or head injury studies. Performance testing demonstrates the system’s repeatability and accuracy in generating impacts. This work contributes to impact research, enabling a broader range of academic and industry groups to develop safer body protection and facilitate its use for further development by the research community.

## Graphical abstract

**Figure:**
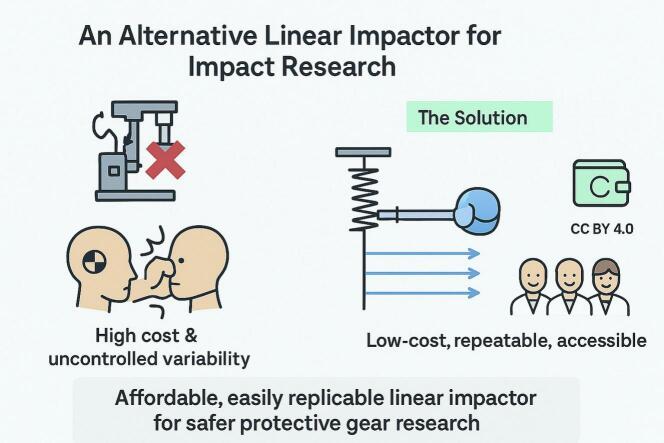

**Specifications table**.

**Table:** 

Hardware name	*Alternative Linear Impactor*
Subject area	Engineering and materials science
Hardware type	Measuring physical properties and in-lab sensors
Closest commercial analog	Pneumatic linear impactor
Open source license	*CC-BY 4.0*
Cost of hardware	2,350 USD
Source file repository	Design files are submitted with the article

## 1 Hardware in context

Head injuries in combat sports, caused by high-force impacts to the skull [1], result in traumatic brain injuries through distinct biomechanical pathways. While linear acceleration primarily causes focal injuries like skull fractures [2], rotational acceleration is the dominant mechanism for diffuse neural damage [3]. This is particularly relevant in combat sports, as it accounts for approximately 90 % of shear stress in brain tissue [4], [5] and is implicated in diffuse axonal injury [6] and traumatic unconsciousness [7]. The critical role of rotational acceleration in injury risk is well-established across various contact sports [8], [9], with boxing impacts generating higher rotational acceleration than American football [10]. This rotational component creates internal strain by inducing rapid angular kinematics that stretch and shear axonal structures [11], [12], and proposed injury thresholds underscore its importance in mild traumatic brain injury [13]. This presents a particularly serious issue for children in combat sports, given their lower injury tolerance compared to adults [14].

Many studies have measured the head's acceleration response to impacts using both direct human measurement and biomechanical surrogates. Head impact measurement devices, whether helmet-mounted [14], [15], [16], [17] or mouthguard sensors [18], have been employed. However, these methods often show limitations in sensitivity, specificity, and overall accuracy for concussion diagnosis. Issues such as helmet-to-head slippage, high error rates, and low specificity in predicting concussive injury compromise the reliability of data obtained from these systems [16], [18]. While approaches using real boxers to impact an instrumented Hybrid III dummy [19] provide valuable data on human-generated impacts, the inherent variability of human input means the impact force cannot be precisely controlled.

To overcome the variability of human input and the limitations of using inputs from human subjects, mechanically controlled impactors offer a promising alternative. Various impact testing methodologies have been engineered to deliver precise and repeatable impacts with adjustable force, velocity, and angular components. These include vertical drop towers [20], [21], pendulum testers [22], linear impactors [23], [24], [25], and rotating impactors [26]. These systems provide a higher degree of control over impact parameters, which is essential for systematic biomechanical studies.

Pneumatic linear impactors are the most common type used in biomechanics and safety testing due to their ability to achieve the high speeds, precise control, and repeatability required by standards like those set by the National Operating Committee on Standards for Athletic Equipment (NOCSAE). However, the high cost of this sophisticated equipment makes it unaffordable for many research labs, even in high-income countries, and prohibitively expensive for institutions in low- and middle-income countries. This study presents an alternative, affordable linear impactor that has demonstrated comparable precision and repeatability, offering a viable solution for testing sport protective equipment in a wider range of resource-limited settings worldwide.

## 2 Hardware description

To overcome the high cost of conventional pneumatic impactors, this study presents a simplified, custom-built linear impactor that does not rely on motors or electrical devices. Instead, our hardware is based on a spring-driven mechanism actuated manually. This design eliminates the need for expensive air compressors and electrical components, reducing both the initial purchase cost and ongoing operational expenses. The system features a custom-machined linear rail and carriage, ensuring smooth motion and precise impact repeatability. This streamlined, modular hardware not only provides a cost-effective solution for protective equipment testing but also serves as a foundational platform for developing further low-cost impact testing devices in the future.

**Fig. 1:**
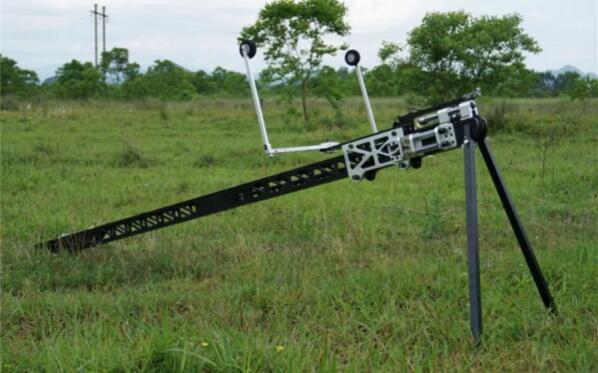
Small UAV sling catapult launcher.

**Fig. 2:**
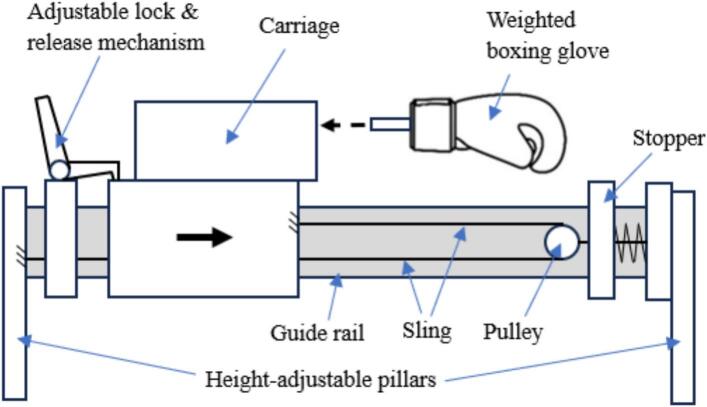
Concept of linear impactor for punching.

The foundation of this apparatus was a modified small unmanned aerial vehicle (UAV) catapult (Fig. 1), repurposed from its original sling mechanism. As required by the NOCSAE standard [27], the impactor should be capable of delivering impacts in the range of 3.0 to 9.0 m/s ± 2 % and be in guided free flight at the time of impact. This range is also designed to cover the critical testing velocities of the NFL Helmet Test Protocol, which typically includes test speeds from 5.5 to 9.3 m/s ± 2 % [28]. The catapult, which has a 2.5-meter rail and a maximum launch capacity of 4.5 kg, was transformed into a custom-built linear impactor designed to deliver controlled and repeatable impacts. This machine was specifically developed to accurately simulate realistic boxing scenarios. The system features a carriage, integrated with the catapult, which holds a standard boxing glove adapted for secure attachment. This assembly serves as the primary impact delivery component. The impact speed is adjusted by altering the carriage's initial release point along the rail via an adjustable lock-and-release mechanism. Upon release, the carriage travels forward, and its motion is arrested by a stopper at the end of the rail. Then, the boxing glove continues its forward trajectory freely after the carriage stops, which ensures an isolated impact profile. The height of the rail can also be adjusted, allowing for precise alignment with various impact locations on the object being tested. The concept of this system is shown in Fig. 2.

## 3 Design files summary

All the design drawings are listed in the Table 1.

**Table 1:** Design files summary.

**Design file name**	**File type**	**Open source license**	**Location of the file**
Assembly Hook sled.pdf	Drawing	CC-BY	Design files are submitted with the article
Assembly slide guide stand	Drawing	CC-BY	Design files are submitted with the article
Assembly Slide Guide	Drawing	CC-BY	Design files are submitted with the article

## 4 Bill of materials summary

The Bill of Materials (BOM) summary presented in Table 2 provides a comprehensive overview of the components required for constructing the custom-built linear impactor, detailing their quantities, costs per unit, total costs, and sources. It is important to note that the costs listed in this table cover only the linear impactor itself. The costs for the stand for the object being tested and other essential equipment for testing, such as a high-speed video camera and lighting, are not included. Additionally, the presented costs are specific to a particular region and may vary due to factors like shipping and taxes, as well as fluctuating over time since the date of purchase.

**Table 2:** Bill of materials summary.

**Designator**	**Component**	**Number**	**Cost per unit USD)**	**Total cost (USD)**	**Source of materials**	**Material type**
Slide rail	Feiyu Tech UAV Catapult for Skywalker X5 & X8 fixed wing aircraft model	1	1,630	1,630 (Include shipping from China to Thailand and import tax)	Alibaba online shop	Metal, plastic, etc.
Supporting stand	Square tubes 1x1 inch, 2.1 mm thick	1	275	275	Local shop	Metal
Lock & launch unit	Assembly Slide Guide	1	165	165	Local shop	Metal
Glove carrier	Assembly Hook sled	1	280	280	Local shop	Metal, plastic

## 5 Build instructions

**Fig. 3:**
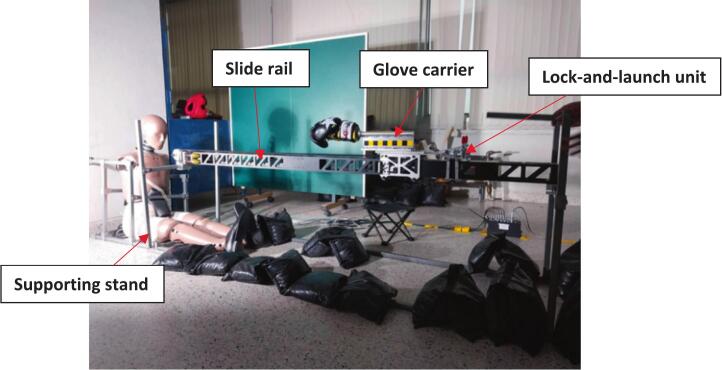
Linear impactor and dummy setup.

The linear impactor consists of four main components: slide rail, supporting stand, lock-and-launch unit, the glove carrier as shown in Fig. 3. Since the slide rail was a pre-existing component from a UAV catapult, the other parts of the apparatus were custom-developed to integrate with it, using the slide rail as the central piece of the design.

### 5.1 Slide rail

The slide rail serves as the central piece of the linear impactor system and comes with a sled, a sling and a sled stopper as shown in Fig. 1. The sling has an approximate spring stiffness of 450 N/m, which can be adjusted by altering its length. All other parts that were not essential for the linear impactor development, such as the front two-leg stand, UAV front wheel guide, wing supporters, and the original lock-and-launch mechanism, were removed.

### 5.2 Supporting stand

**Fig. 4:**
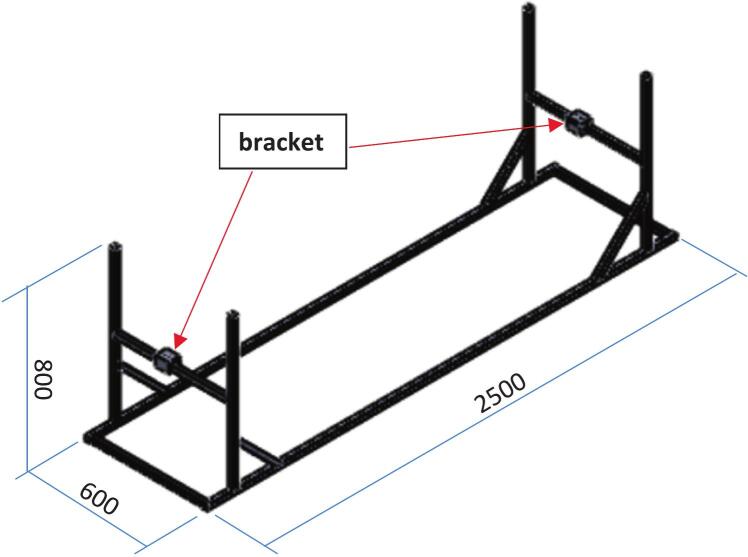
Supporting stand.

**Fig. 5:**
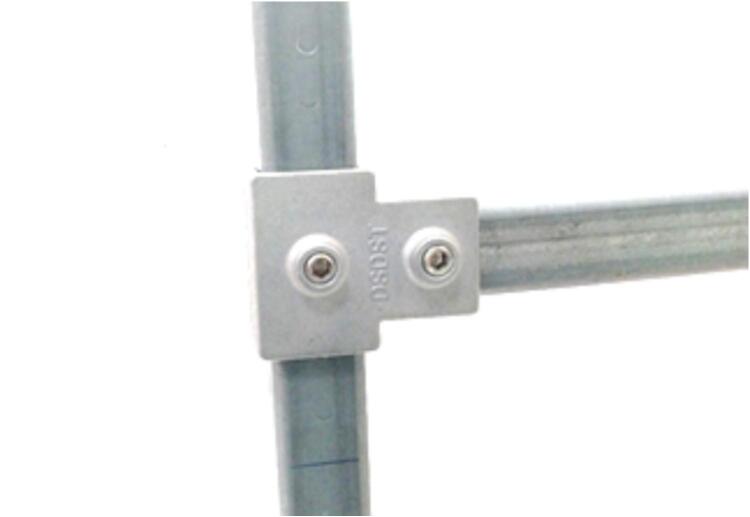
Steel coupler for height adjusting.

The supporting stand, with overall dimensions of 2500 x 600 x 800 mm, is the foundational component that supports and anchors all other parts of the linear impactor as shown in Fig. 4. Constructed from 1x1 inch square tubes with a 2.1 mm wall thickness, the stand features four posts and two horizontal beams with brackets that are fixed to both ends of the slide rail, allowing the rail to be adjusted in a lateral direction. To allow for height adjustments, steel couplers were integrated into the design as shown in Fig. 5, enabling the rail's height to be varied and aligned with different impact locations on the tested object.

### 5.3 Lock-and-launch unit

**Fig. 6:**
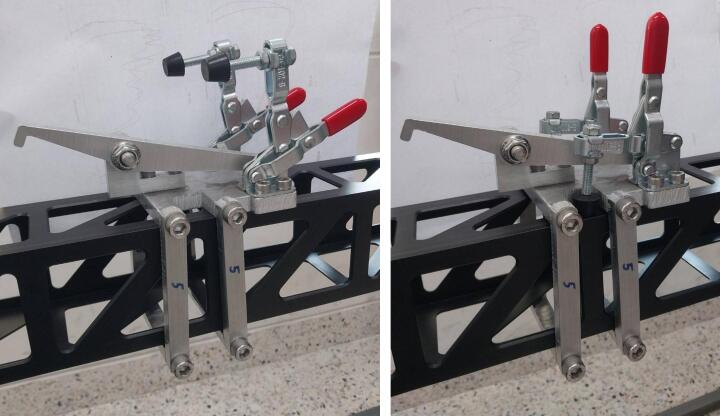
Lock-and-launch unit; no position lock (left) and position lock (right).

The lock-and-launch unit is designed to secure and release the sled, which is an integrated part of the slide rail system as shown in Fig. 6. This unit can be moved and fixed at any position along the rail to adjust the impact speed. The unit uses toggle clamps to achieve reliable position locking.

### 5.4 Glove carrier

**Fig. 7:**
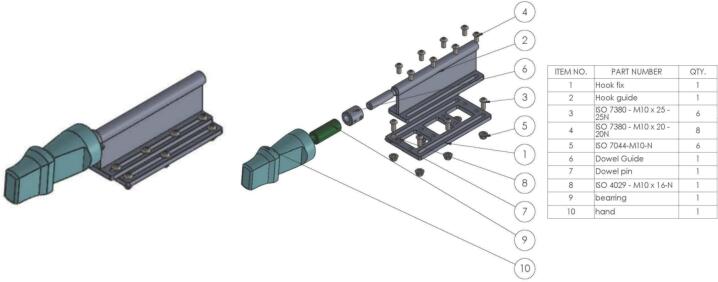
Glove carrier.

The glove carrier is designed to carry the boxing glove that impacts the object after the sled is released. It consists of two main parts: a carrier base and an artificial fist. The carrier base is made to fix securely to the sled and allows for the adjustment of the glove's longitudinal position. A dowel on the top of the carrier base is used to plug the artificial fist onto the base. The artificial fist itself is made from a 3D-printed material to imitate the human fist and wrist in a straight punch position and has an internal linear bearing and a dowel guide insert as shown in Fig. 7. This assembly allows the artificial fist to fly freely at the time of impact, as required by NOCSAE, once the sled hits the stopper. The weighted boxing glove is worn on the artificial fist during testing. This artificial fist can also be interchanged with other configurations to meet the user's specific needs.

## 6 Operation instructions

**Fig. 8:**
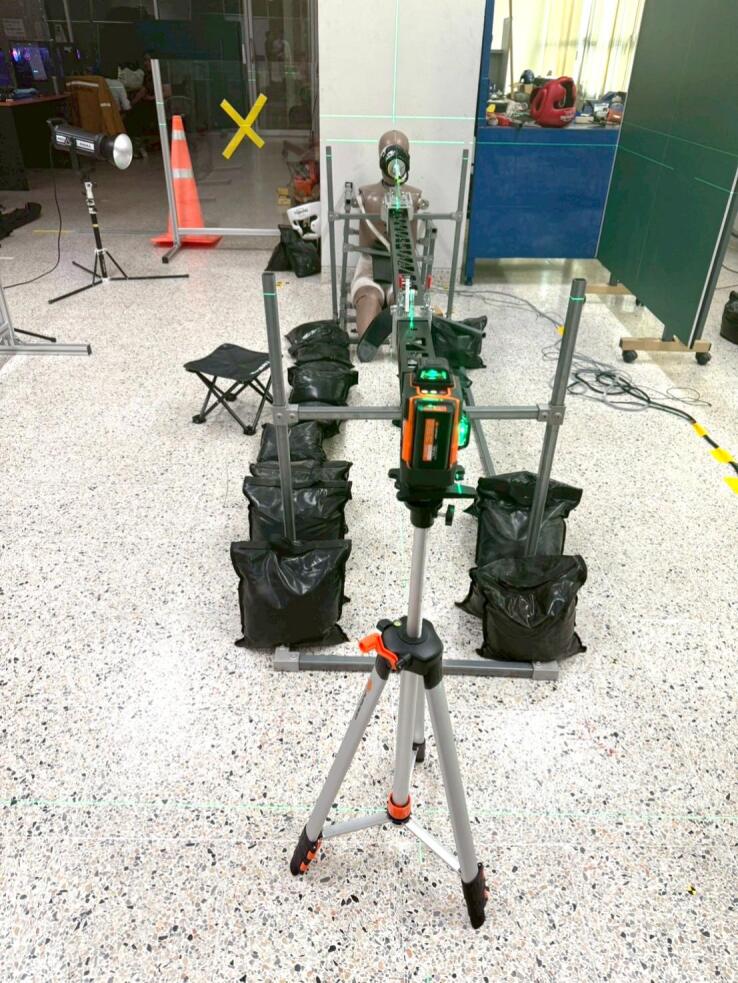
Use of marking laser pointer to specify impact location.

**Fig. 9:**
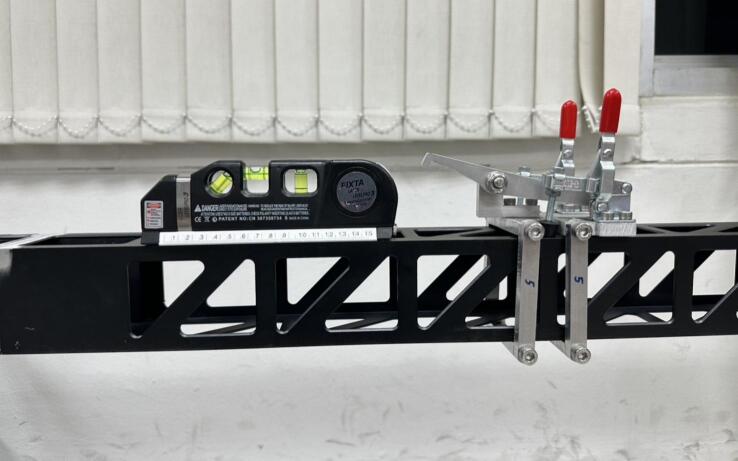
Use of spirit level to confirm horizontal.

Before using the linear impactor, a series of preparations must be completed. First, the precise impact location on the object must be selected. Once the location is specified, the slide rail must be adjusted to meet it in both the lateral direction and height. A marking laser pointer is used to confirm the lateral alignment at both ends of the slide rail, ensuring it is perfectly aimed at the target as shown in Fig. 8. The height of the slide rail is adjusted using steel couplers on all four posts, and a spirit level—either analog or digital—is used to confirm that the rail is perfectly horizontal as shown in Fig. 9.

After confirming the impact location and rail level, sandbags are placed on the supporting stand's base to prevent the entire system from moving when the sled hits the stopper. The weighted glove and artificial fist assembly is then plugged into the carrier. The lock-and-launch unit is fixed at the position that will generate the required impact speed. The sled is manually pulled back and locked into place with the lock-and-launch unit. When ready, the user presses the lever on the lock to release the sled, initiating the impact sequence.

## 7 Validation and characterization

### 7.1 Impact speed and free-flight validation

To verify the accuracy and precision of the linear impactor's speed control, a test was set up. The setting speeds were 2.9, 4.1, 5.0, 6.2, 8.0, 8.3, and 10.0 m/s, with each impact repeated 10 times. The impact speeds were measured using a high-speed Phantom v2512 camera, which operated at 3,000 frames per second. Frame-by-frame analysis with Photron FASTCAM viewer software enabled a precise measurement of velocity at the moment of impact. Reflective markers placed on the glove and carrier aided in the speed calculation. Spatial measurements from the video had an estimated accuracy of ± 1–2 mm, which was based on a prior calibration with a known scale bar.

The results of speed verification are shown in Table 3. The custom-built linear impactor demonstrates a high degree of both accuracy and precision in its speed control across the tested range. The accuracy of the machine is excellent, as the mean impact speed for each test is very close to the required speed. The maximum error is consistently low, ranging from a high of 0.86 % at 2.9 m/s down to a minimum of −0.89 % at 10.0 m/s. This performance indicates that the machine reliably hits its target velocity, staying well within the industry specifications (such as NOCSAE and NFL), which typically require an impactor to be capable of propelling the mass to the specified velocity within ± 2 %. The system also exhibits strong precision, as evidenced by the low variation in the mean speeds across each set of ten tests. The consistency of these results, as indicated by the small margins of error, confirms that the impactor is capable of delivering highly repeatable impacts essential for rigorous testing.

**Table 3:** Impact speed accuracy and precision.

**Setting speed (m/s)**	**Mean actual impact speed (m/s)**	**SD** **(m/s)**	**Coefficient of variation (%)**	**Maximum error (%)**
2.9	2.92	0.01	0.37	0.69
4.1	4.08	0.02	0.38	−0.89
5.0	5.03	0.01	0.15	0.86
6.2	6.23	0.01	0.17	0.65
8.0	8.03	0.02	0.18	0.55
8.3	8.32	0.01	0.08	0.40
10.0	9.99	0.02	0.15	−0.20

**Fig. 10:**
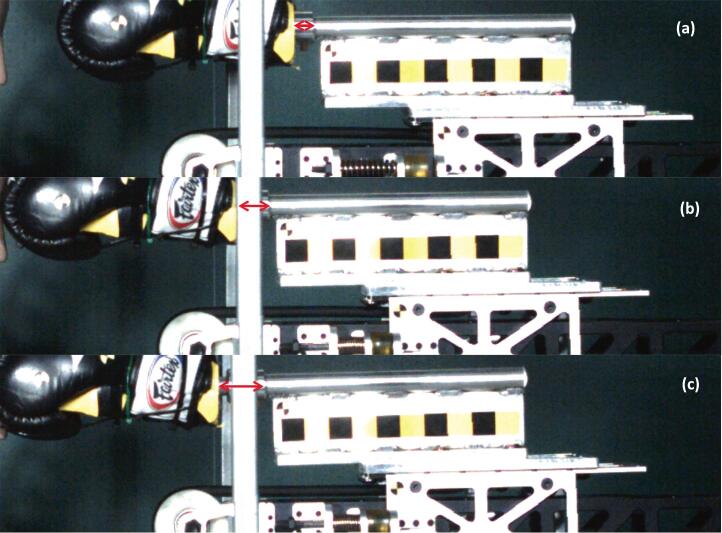
High-speed video sequence demonstrating the carriage arrest and subsequent free-flight of the boxing glove: (a) the carriage immediately prior to contacting the rubber-bumpered stopper, (b) the carriage fully arrested by the stopper, and (c) the boxing glove assembly continuing in guided free-flight toward target.

Fig. 10 illustrates the mechanism that enables the guided free-flight of the boxing glove. Upon release, the carriage is propelled by spring tension along the slide rails until it impacts the rubber-bumpered stoppers at the end of the track. During this arrest phase, the carriage is rapidly decelerated to a complete stop, while the boxing glove assembly—supported by independent linear bearings—continues its forward trajectory via inertia. This mechanical decoupling is clearly demonstrated in Fig. 10 by the visible gap between the dowel guide and the boxing glove assembly (indicated by the red arrow). This separation ensures that the glove maintains its alignment while traveling independently of the carriage, resulting in a purely inertial impact that is free from secondary mechanical interactions or propulsion-related 'edge effects'.

### 7.2 Dummy head response characterization

The linear impactor was validated with a full-body 50th percentile male Hybrid III dummy to verify variation of dummy head responses. The dummy was securely mounted using a rigid metal support structure (Fig. 3) to ensure repeatability and minimize extraneous motion. Impacts were measured using a triaxial array of three uniaxial accelerometers (Kyowa model ASE-A-1KM32Z7L) mounted on a triaxial mounting block and a three-axis angular rate gyro (Kyowa model GSAT-A-900), both rigidly mounted at the head's center of gravity (CG) by being directly bolted to the internal aluminum skull structure. Each accelerometer measured linear acceleration along a single primary axis (in m/s2), while the rate gyro simultaneously measured angular velocity across three orthogonal axes (in deg/s). Rotational acceleration was derived through the differentiation of the angular velocity data. All raw sensor data were acquired using a high-speed data acquisition system at a sampling rate of 10 kHz with a SAE J211 CFC 1000 filter.

All sensors were factory-calibrated and the mounting block was constructed of lightweight aluminum. The total added mass of the triaxial array, gyro, and mounting block was 29 g. This added mass is less than 0.64 % of the total Hybrid III head mass (4.5 kg), ensuring that the inertia properties of the head form were not significantly altered. The sensors and mounting block were carefully positioned to ensure the combined center of gravity remained within ± 1 mm of the standard Hybrid III head CG. The rigid, bolted attachment ensured no relative motion between the sensor package and the skull, guaranteeing accurate measurement of the head's rigid-body motion.

**Fig. 11:**
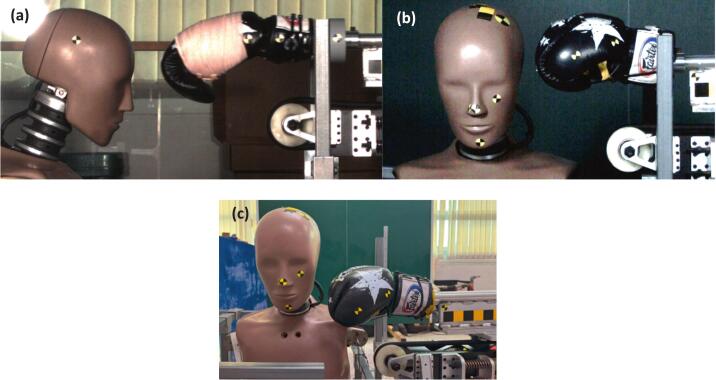
Impact locations on dummy’s head; a) forehead, b) temple, and c) jaw.

The same high-speed camera and software were used to verify the impact speed at the moment of contact, with reflective markers placed on the dummy head's CG and on the boxing glove aiding in speed calculation. The impact speed was set at 6.2 m/s with a corresponding impacting glove mass of 1.9 kg. Three distinct impact locations on the dummy head were selected to represent common strike targets in boxing; forehead, temple, and jaw, as depicted in Fig. 11. Three repetitions were performed for each impact location. Following each impact to the dummy head, the linear accelerations generated by the linear impactor were recorded for each location.

**Fig. 12:**
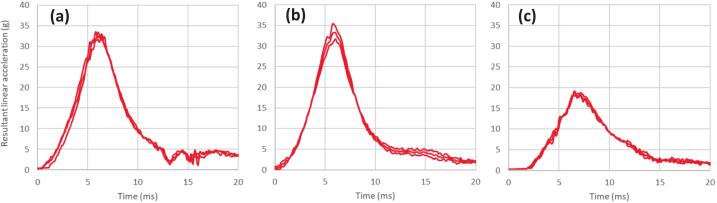
Resultant linear acceleration time histories for each impact location; a) forehead, b) temple, and c) jaw.

**Fig. 13:**
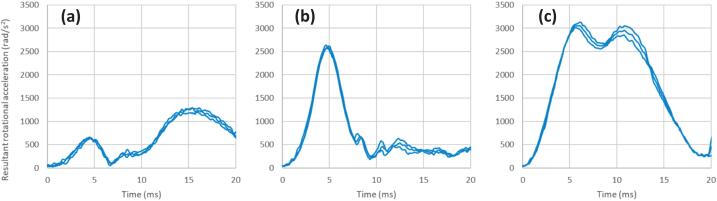
Resultant rotational acceleration time histories for each impact location; a) forehead, b) temple, and c) jaw.

Fig. 12, Fig. 13 present the linear and rotational acceleration time histories for the headform responses at each impact location. The results demonstrate high repeatability across all test conditions, as evidenced by the tight clustering of the multiple trial curves. The individual profiles for these impacts are nearly indistinguishable, confirming that the system produces highly consistent outcomes under identical experimental parameters. This high degree of precision validates the reliability of the measurement chain when pairing the custom linear impactor with the Hybrid III dummy. Furthermore, the PLA time histories in Fig. 12 show a clean return to baseline following the primary peak, confirming the absence of secondary impacts or carriage interference during the glove-to-head interaction.

Table 4 presents the peak kinematic responses of the Hybrid III headform at an impact velocity of 6.2 m/s, demonstrating high repeatability across three distinct anatomical locations: the forehead, temple, and jaw. For linear acceleration, the system achieved a high degree of precision with coefficients of variation (CV) as low as 1.5 % at the jaw, although a slightly higher CV of 5.7 % was observed at the temple, likely due to the complex surface geometry of that region. Rotational acceleration metrics similarly show strong consistency, with the jaw and temple locations exhibiting CV values of 1.9 % and 1.3 %, respectively, while the forehead recorded a CV of 3.9 %. Collectively, these small standard deviations and low CV values across both linear and rotational domains confirm that the custom-built linear impactor reliably delivers consistent impact energy, satisfying the requirements for repeatable biomechanical head injury research.

**Table 4:** Peak linear and rotation accelerations response of Hybrid III dummy’s head at impact speed of 6.2 m/s on various locations.

**Location**	**Peak linear acceleration (g)**	**SD** **(g)**	**Coefficient of variation (%)**	**Peak rotational acceleration (rad/s^2^)**	**SD** **(rad/s^2^)**	**Coefficient of variation (%)**
Forehead	33.1	0.7	2.0	1262.2	48.8	3.9
Temple	33.5	1.9	5.7	2599.2	34.0	1.3
Jaw	18.6	0.3	1.5	3068.6	59.7	1.9

This preliminary assessment of impact locations highlights that the specific site of impact significantly dictates the resulting Peak Linear Acceleration (PLA) and Peak Rotational Acceleration (PRA), with the results indicating that different regions produce distinct kinematic signatures. The findings demonstrate that while frontal impacts to the forehead primarily result in high linear responses, impacts to the temple and jaw locations generate substantially higher rotational components; notably, the jaw produced the highest PRA (3068.6 rad/s2) despite having the lowest linear response (18.6 g). These results are consistent with established biomechanical literature, which posits that impacts further from the head's CG—such as those to the mandible—create a larger moment arm that facilitates increased angular motion [10], [19]. This variation mirrors observations in studies of professional sports impacts where lateral or “chin” strikes are frequently linked to higher rotational injury metrics compared to direct forehead strikes. By reliably capturing these site-specific differences with high precision, the linear impactor is validated as an effective tool for replicating the complex loading conditions required to evaluate brain injury criteria across various real-world scenarios.

### 7.3 Capabilities of the hardware

• Impact speed: 2.9 – 10 m/s ± 1 %
• Impact mass: up to 4.5 kg
• Do not need air pressure or electricity to operate
• Overall dimensions: 2500 x 600 x 800 mm
• Total weight: 28 kg (without impact mass)
• Free flight of impact mass at the time of impact
• This linear impactor can be paired with full body anthropometric test devices (ATDs) or head and neck assembly ATDs with a stand or any object being test

### 7.4 Safety considerations

• Due to the high tension generated by the spring mechanism at velocities above 5 m/s, manual loading of the carriage requires significant force. To mitigate operator strain and ensure safety, we recommend that two operators be utilized when manually pulling the carriage to the maximum required launch position.
• The current lock-and-release mechanism utilizes a simple lever that requires remote activation to ensure operator safety during high-velocity launches. During testing, this lever was depressed using a non-hand-operated tool, for example, a stick. For all future iterations, we recommend a revised design utilizing a remote trigger (e.g., a cable or rope) to maximize the distance between the operator and the system at the time of launch.
• Given that the impact mass enters free flight immediately before impact, there is a risk of the mass bouncing or deviating unpredictably post-impact. We recommend installing robust safety barriers or fences around the test area (e.g., the instrumented dummy) to contain any unexpected debris or bouncing components.

### 7.5 Limitations of the hardware

• The system's current launch capacity represents a significant limitation regarding its potential use for official football helmet certification. The catapult system used in this study was fundamentally designed to accurately simulate the mass and velocity of an actual punch in realistic boxing scenarios. This is incompatible with the required total impactor mass of 15.6 kg for the pneumatic linear impactor used in football helmet testing. This discrepancy is a direct result of the UAV catapult's 4.5-kg launch limit. However, the successful demonstration of this concept—achieving the required velocity range (2.9 to 10 m/s)—shows that the design principle is effective. Future iterations of this concept could utilize a higher-capacity linear propulsion UAV catapult to accommodate the necessary 15.6-kg impact mass, thereby addressing the high-mass requirements of football helmet certification.
• Although the impact speed has proven its repeatability, it's still recommended that a speed measurement should be re-verified every time the machine is used or every 200 cycles. This is because unexpected errors can occur due to its purely mechanical components, such as slight changes in the spring's elasticity or wear and tear on the linear rail over time. Users should perform a 'calibration fire' without a target and measure the velocity by any available speed measurement equipment. If the measured velocity deviates by more than 2 % from the established baseline for that compression distance, the user should adjust the position of Lock-and-launch unit or sling-end bracket to compensate for the loss in stiffness or replace the sling itself. Additionally, a visual inspection of the sling's free length should be conducted; a reduction in length indicates permanent deformation (spring set), necessitating replacement to ensure kinematic consistency.

## CRediT authorship contribution statement

**Poomkarn Taedullayasatit:** Writing – original draft, Visualization, Methodology. **Sitthichok Sitthiracha:** Writing – review & editing, Validation, Supervision, Methodology, Formal analysis, Data curation, Conceptualization. **Manus Dangchat:** Software, Investigation. **Nattawood Prasartthong:** Investigation.

## Declaration of competing interest

The authors declare that they have no known competing financial interests or personal relationships that could have appeared to influence the work reported in this paper.
